# Pharmacogenetics of *ugt* genes in North African populations

**DOI:** 10.1038/s41397-018-0034-4

**Published:** 2018-07-31

**Authors:** M. Gaibar, A. Novillo, A. Romero-Lorca, M. E. Esteban, A. Fernández-Santander

**Affiliations:** 1 Biomedical and Health Sciences Faculty, Universidad Europea de Madrid, Tajo s/n, 28670 Villaviciosa de Odón, Madrid Spain; 2 Faculty of Biology, University of Barcelona, Diagonal 645, 08028 Barcelona, Spain; 3 Institut de Reçerca de la Biodiversitat (IRBio), University of Barcelona, Barcelona, Spain

## What we already know

Cytochrome P450 (CYP450), sulfotransferase (SULT), and glucuronidase (UGT) enzymes play roles in the phase I and phase II metabolism of most clinically prescribed drugs. As polymorphisms in these genes may alter enzyme activities, most prescribed drugs will differ in their efficacy and side effects. In prior work, we showed that besides polymorphisms in *CYP450*, those in *SULT* and *UGT* also give rise to different serum levels of some drug metabolites than detected in wild-type carriers of the genes [1]. To date, most pharmacogenetic studies have examined Asian and Caucasian populations and although the pharmacogenetics of *CYP450* genes has been explored in sub-Saharan countries, scarce data exist for African genetic variations in *SULT* and *UGT* [2, 3].

Africans show an extremely high incidence of malaria, tuberculosis, and HIV/AIDS, along with a growing rate of noncommunicable diseases, especially diabetes, and hypertension [4]. Data concerning these diseases and others indicate a wide diversity of prevalence and mortality rates among African countries. Although therapeutic drugs have improved life expectancies in many countries, most drugs have been associated with adverse drug reactions (ADRs) [4]. As CYP450 enzymes metabolize antiretrovirals, antimalarials, and antipsychotics, knowledge of their genetic variability could be useful for clinicians. In some developed countries, a high economic burden of deaths has been attributed to ADRs whereas no such estimates exist for African populations. This lack of data highlight a need to improve knowledge on ADRs in Africa, mainly when these effects are associated with drugs used to treat the main killer diseases in this continent. Knowledge such as this is essential to develop and implement pharmacogenetic tests both in developing and developed countries.

Africans show the broadest genetic variability of all human populations. This is because the African origin of mankind has meant a longer time period for genetic diversity in Africans compared to non-Africans. For example, according to known CYP450 variability, 90% of clinically relevant *CYP2C8*, *CYP2C19*, and *CYP2D6* variants occur within the range of 0–24% [5]. North African ethnicities have been largely overlooked regarding the detection of genes involved in drug metabolism such as SULTs and UGTs. Besides their genetic variability, African populations are characterized by their remarkable linguistic and cultural diversity, consistent with the huge diversity of landscapes and habitats of this continent. Current North African cultures (from Morocco to Egypt) have an ancient Berber background with influences from several civilizations of different historical times. The Arabs settled permanently in North Africa and although they persuaded Berbers to adopt Islam, some large Berber groups in Morocco, Algeria, and Tunisia have retained their Berber language and customs and avoided mixed marriages until today. Prior studies of the genetic variability of these North African groups have detected vast genetic heterogeneity and a lack of genetic groupings by either geographical or linguistic criteria [6]. This considerable genetic variability has been previously reported for *CYP3A4*, *CYP3A5*, *SULT1A1, SULT1A2*, and *SULT1E1* in North African populations [7].

Here, we stress the importance of assessing the frequencies of CYPs, SULTs, and UGTs in North African populations because of their role in the metabolism of many drugs and their association with various types of cancer. In ongoing work, we have been looking at the UGT mutations *UGT1A4* Pro24Thr (rs6755571), *UGT1A4* Leu48Val (rs2011425), *UGT2B7* His268Tyr (rs7439366), *UGT2B15* Asp85Tyr (rs1902023), *UGT2B15* Lys523Thr (rs4148269), and *UGT2B17*del in populations from Morocco, Libya, Tunisia, and Algeria. Some variants seem to confer reduced UGT activity, for instance, the UGT2B15^85Tyr^ allele in response to oxazepam and lorazepam and UGT1A4^48Val^ and UGT2B7^268Tyr^ with effects on tamoxifen metabolism [8, 9]. In contrast, no effects of the UGT2B17 deletion have been detected on oxazepam and androgenic steroid substrate availability, perhaps owing to the possible duplication event origin of UGT2B15 and UGT2B17, which show similar sequence identity [10].

## North African populations

The groups examined in this work underway comprise the general population representative of two Moroccan Berber localities (Asni region in the High Atlas and Sidi Bouhria in the northeast Atlas), one Algerian Berber region and different areas of Tunisia and Libya. Four hundred and eighty-four DNA samples—collected from healthy, unrelated individuals (both sexes) aged 18–60 years and native to the regions they lived in (at least three generations)—were processed as previously described [7]. All subjects signed an informed consent form approved by the ethics committees of the Universities responsible for sample collection (Chouaib Doukkali University in Morocco, Monastir University in Tunisia, and Abderrahmane Mira Bejaia University in Algeria). Individuals from Tunisia and Libya were Arabic speaking, whereas the Moroccan and Algerian subjects sampled spoke Berber.

## UGT polymorphisms

The allelic variants *UGT1A4*^24Thr^, *UGT1A4*^48Val^, *UGT2B7*^268Tyr^, *UGT2B15*^85Tyr^, *UGT2B15*^523Thr^, and *UGT2B17*^del^ were genotyped as described in Romero-Lorca et al. [9] using conventional quantitative polymerase chain reaction and polymerase chain reaction-restriction fragment length polymorphism techniques. Allele frequencies were estimated through direct gene counts. Hardy–Weinberg equilibrium was assessed using an exact test. North African data were compared with data available for European and Sub-Saharan African populations from the 1000Genomes database (http://www.1000Genomes.org) using the pairwise population differentiation test included in Arlequin v.3.5 (http://cmpg.unibe.ch/software/arlequin35/). Genetic diversities were estimated using Nei’s formula [11].

Allele frequencies of the *UGT* polymorphisms are provided in Table 1. All genes examined were in Hardy–Weinberg equilibrium. Compared with European and Sub-Saharan African samples, the Moroccan samples show higher *UGT1A4*^*48Val*^ and *UGT2B7*^*268Tyr*^ allele frequencies (0.260 and 0.464, respectively, Table 1). Pairwise population differences highlight differentiation between the two Nigerian samples and North Africans and Europeans for all markers except *UGT1A4*^*48Val*^. With regard to the internal heterogeneity of North African samples, Libya was the population showing the lowest average genetic diversity (0.242), whereas northeast Atlas Morocco showed the highest diversity (0.373). Greatest population differentiation was observed for *UGT1A4*^48Val^, *UGT2B7*^268Tyr^, and *UGT2B17*^*del*^, mainly owing to their frequencies in northeast Atlas Moroccans, Tunisians, and Libyans. These results are in agreement with our published data for *CYP* and *SULT* polymorphisms, which also largely exceeded the variation ranges described for European populations [7].

**Table 1:** Allele frequencies of *UGT1A4* Pro24Thr, *UGT1A4* Leu48Val, *UGT2B7* His268Tyr, *UGT2B15* Asp85Tyr, *UGT2B15* Lys523Thr, *UGT2B17*del polymorphisms in five North African populations

Polymorphism SNP reference	High Atlas Morocco (95)	Northeast Atlas Morocco (94)	Algeria (83)	Tunisia (104)	Libya (108)	Iberians (107)	Italians (107)	British (91)	Yoruba (99)	Esan (108)
*UGT1A4*^*24Thr*^ rs6755571	0.057	0.052	0.054	0.027	0.067	0.042	0.051	0.060	0.000	0.010
*UGT1A4*^*48Val*^ rs2011425	0.080	0.260	0.048	0.025	0.144	0.089	0.159	0.071	0.083	0.101
*UGT2B7268Tyr* rs7439366	0.464	0.250	0.372	0.086	0.186	0.450	0.520	0.530	0.210	0.240
*UGT2B15*^*523Thr*^ rs4148269	0.605	0.500	0.518	0.576	0.558	0.621	0.617	0.648	0.106	0.136
*UGT2B15*^*85Tyr*^ rs1902023	0.430	0.452	0.415	0.357	0.394	0.514	0.495	0.456	0.569	0.581
UGT2B17del	0.039	0.315	0.072	0.174	0.244	No data available in 1000Genomes project				

Data from the 1000Genomes project are also provided for European population groups (Iberians, Italians, and British) and for African samples from Nigeria (Yoruba in Ibadan and Esan). Sample sizes indicated between parentheses

## Associated risks or functional roles

The association between some gene polymorphisms and the presence of functional role- or risk associated phenotypes is a relevant issue when studying such diverse groups as North African populations. Given marked differences in CYP, SULT, and UGT polymorphisms between Caucasian and Sub-Saharan African groups and their relevance both in drug pharmacogenetics and risks of various types of cancer, detailed study of the frequencies of these mutations is needed to improve clinical decision-making in African healthcare institutions. In prior work, we detected a 2–3 times higher frequency of CYP3A4*1B associated with prostate cancer in North Africans compared with European Caucasians (Table 2), peaking at 29% in Moroccan Berbers [7]. Some SULT gene alleles are also important because of both their link to endometrial cancer and their high frequency in some areas of North Africa. For instance, the SULT1A1*2 allele appears in ~ 50% of northeast Atlas Moroccan Berbers (Table 2) [7].

**Table 2:** Functional role and risk associated genotypes in North African populations

Polymorphism SNP reference	Genotype	Associated functional role/risk	Allele frequency range in North African populations	Ref.
CYP3A4*1B rs2740574	Carriers of allele *1B	Allele *1B related to reduced CYP3A4 gene expression Prostate cancer in African Americans	11–29 %	[7]
SULT1A1*2 rs9282861	Carriers of allele *2	Allele *2 related to lower heat stability and activity of the enzyme Endometrial cancer in European Caucasians	10–47 %	[7]
SULT1E1*2 rs3736599	Carriers of allele *2	Allele *2 related to a reduction in enzyme activity of ~ 90% Endometrial cancer in European Caucasians and African Americans	6–16 %	[7]
UGT1A448Val rs1902023	Homozygous wt/wt allele (48 Leu)	wt allele: normal enzyme activity UGT1A4Leu/Leu better efficacy of lamotrigine (epileptic drug used in pediatric patients)	2–26 %	[12]
UGT2B1585Tyr rs1902023	Carriers of 85Tyr Homozygous 85Tyr	Allele 85tyr related to lower glucuronidation activity − Possibly reduced prostate cancer risk: high-activity allele, increased rates of androgen glucuronidation, lower intraprostate androgen levels. − Mean apparent oral oxazepam clearance significantly lower than in wt/wt patients	35–45%	[13, 14]
UGT2B17del	Homozygous del/del	Null allele: complete loss of UGT2B17 activity − Increased risk of lung adenocarcinoma in women: associated with decreased NNAL (tobacco-specific metabolite of nitrosamine) glucuronidation rates. − Reduced risk of colorectal cancer: associated with higher levels of antioxidant protective dietary components.	4–31%	[16, 17]

Given these marked ethnic differences in CYP and SULT genes and their association with drug pharmacogenetics and disease risk, UGT gene polymorphisms must also be considered in population studies because of their known influence in the metabolism of some drugs such as lorazepam and tamoxifen [8, 9]. The higher frequency of the UGT1A448Val mutation (26% in some Moroccan areas; Table 1) relative to those reported in Caucasians is especially remarkable. Indeed, the wt homozygous genotype UGT1A4Leu/Leu has been linked to a better efficacy of lamotrigine (epileptic drug used in pediatric patients) [12]. The UGT2B1585Tyr allele, which appears in up to 45% of individuals in some North African areas, is a high-activity allele possibly associated with a reduced prostate cancer risk according to increased rates of androgen glucuronidation leading to lower intraprostate androgen levels (Table 2) [13]. It has also been reported that patients homozygous for the UGT2B1585Tyr mutation show a significantly lower mean apparent oral oxazepam clearance rate compared with wt/wt patients [14]. The UGT2B17 allele deletion appeared at a rate worth considering though within the reported range for other populations despite the variation observed among ethnic groups, e.g., rates of 21% and 33%, respectively, described for African Americans and Caucasians [15]. Women with both copies of the UGT2B17 gene deletion show an increased risk of lung adenocarcinoma associated with decreased NNAL (tobacco-specific metabolite of nitrosamine) glucuronidation rates [16]. Further, a reduced risk of colorectal cancer has been described in Caucasian men homozygous for UGT2B17del [17]. This last link has been related to the preferred metabolism of UGT2B17 over that of certain nonsteroidal anti-inflammatory drugs and over flavonoids with antioxidant properties, such that individuals lacking UGT2B17 may have higher levels of these protective dietary components [17].

The variation ranges of CYP, SULT, and UGT genes observed in the North African populations are considerably higher than those reported for European Caucasians. Given the known links of some of these polymorphisms both with ADRs and the risk of some cancer types, studies are urgently needed to improve knowledge of the prevalence of these allelic variants. Future studies are also required to examine the impact of these genetic variants on different drugs in terms of their availability, metabolism, and efficacy. This will be a good starting point to develop pharmacogenetic tests for use in clinical practice that will avoid unnecessary costs to healthcare systems. The information arising from these studies will have useful implications for healthcare professionals both in developing countries and in other countries when managing patients of North African origin.
